# The Multifaced Perspectives of Genetic Testing in Pediatric Cardiomyopathies and Channelopathies

**DOI:** 10.3390/jcm9072111

**Published:** 2020-07-04

**Authors:** Nicoleta-Monica Popa-Fotea, Cosmin Cojocaru, Alexandru Scafa-Udriste, Miruna Mihaela Micheu, Maria Dorobantu

**Affiliations:** 1 Department of Cardiology, Clinical Emergency Hospital of Bucharest, Floreasca Street 8, 014461 Bucharest, Romania; fotea.nicoleta@yahoo.com (N.-M.P.-F.); cojocaru.r.b.cosmin@gmail.com (C.C.); alexscafa@yahoo.com (A.S.-U.); maria.dorobantu@gmail.com (M.D.); 2 Department 4—Cardiothoracic Pathology, University of Medicine and Pharmacy Carol Davila, Eroii Sanitari Bvd. 8, 050474 Bucharest, Romania

**Keywords:** pediatrics, cardiomyopathies, channelopathies, genetic testing, psychological impact

## Abstract

Pediatric inherited cardiomyopathies (CMPs) and channelopathies (CNPs) remain important causes of death in this population, therefore, there is a need for prompt diagnosis and tailored treatment. Conventional evaluation fails to establish the diagnosis of pediatric CMPs and CNPs in a significant proportion, prompting further, more complex testing to make a diagnosis that could influence the implementation of lifesaving strategies. Genetic testing in CMPs and CNPs may help unveil the underlying cause, but needs to be carried out with caution given the lack of uniform recommendations in guidelines about the precise time to start the genetic evaluation or the type of targeted testing or whole-genome sequencing. A very diverse etiology and the scarce number of randomized studies of pediatric CMPs and CNPs make genetic testing of these maladies far more particular than their adult counterpart. The genetic diagnosis is even more puzzling if the psychological impact point of view is taken into account. This review aims to put together different perspectives, state-of-the art recommendations—synthetizing the major indications from European and American guidelines—and psychosocial outlooks to construct a comprehensive genetic assessment of pediatric CMPs and CNPs.

## 1. Introduction

Inherited cardiomyopathies (CMPs) and channelopathies (CNPs) are cardiac disorders with a very heterogenic presentation and evolution from asymptomatic gene carriers to heart failure development, malignant arrhythmias or sudden cardiac disease (SCD). Following a complex continuum from genetic mutations to clinical manifestations [1], CMPs and CNPs can be early detected with the implementation of lifesaving strategies when genetic testing is performed. Pediatric CMPs and CNPs, although rare, represent a serious condition, remaining a leading cause of death in children. In previously healthy children, conventional autopsy of adolescents with SCD fails to establish the diagnosis in the absence of genetic analysis [2], as certain CMPs and CNPs do not display structural abnormalities. For better management for these patients, etiology-specific treatment is needed, based mainly on a comprehensive evaluation, including genetic analysis, as this increases the accuracy of the etiological diagnosis, with consequences for treatment, evolution and family genetic counselling. In children, predictive genetic testing (i.e., testing an apparently healthy relative in order to predict future risk of disease) is debatable and should be individualized, case-by-case, due to the possible psychological impact [3,4]. However, the age to start the genetic testing in asymptomatic family members depends largely on the disease. For diseases such as long QT syndrome (LQTS) and catecholaminergic polymorphic ventricular tachycardia (CPVT), where there are clear strategies to implement able to prevent SCD, mutation-specific genetic testing is highly advisable and should be performed independent of age. For others, it is reasonable to monitor for the manifestation of the disease, rather than carrying out genetic testing for a condition that may never develop.

The present review aims to show two different perspectives of genetic diagnosis in the pediatric population with CMPs and CNPs, including state-of-the art guideline recommendations and the point of view of the family.

## 2. Guidelines Perspective

### 2.1. Cardiomyopathies

CMPs are rare in the pediatric population with a prevalence of 1–2/100.000 [5], with lower incidence in Finland (0.74/100.000) [6]. These can be isolated or part of a systemic disease, an essential feature to be clarified from the early beginning as this may influence the management of the disease. Although pediatric and adult CMPs share the same morphological characteristics, the cause, evolution and outcome are largely different. What is particular in CMPs developed at pediatric age, especially those occurring in the first year of life, is the high likelihood of an underlying metabolic or mitochondrial cause. The genetic mutations involved in pediatric CMPs are extremely heterogenous as these can be due to inborn metabolic disorders or neuromuscular or genetic syndromic conditions. Thus, the genetic testing in pediatric subjects is difficult due to the comprehensive panel of genes to be included, as the CMP can be only one manifestation of the disease, not entirely displayed due to early age. Moreover, the difficulty also comes from the fact that most of the data and literature also mentioned herein refer to the adult population, and only a minority of studies concentrate on the pediatric counterpart.

Pediatric CMPs have been classified, based on morphology, into five categories: hypertrophic cardiomyopathy (HCM), dilated cardiomyopathy (DCM), restrictive cardiomyopathy (RCM), arrhythmogenic cardiomyopathy (ACM) and left ventricular non-compaction (LVNC). A remark should be made here regarding the addition of the genetic cause to the phenotypic description, recommended by the American College of Cardiology/American Heart Association (ACC/AHA), mainly referring to the HCM group, which includes only those with sarcomere mutations [7].

Next-generation sequencing-based studies have demonstrated that CMPs are highly genetically heterogeneous with many of these mutations “private”, found only in single families, making the discrimination between a pathogenic and a rare variant with no clinical significance extremely challenging [8]. The genetic testing can have three resolutions: positive (identifies a pathogenic (P)/likely pathogenic (LP) variant), negative (lack of P/LP variant) or inconclusive (identifies a variant with unknown significance (VUS)). The identification of a VUS does not imply the variant is not disease causing but that data is not comprehensive enough to enable a definite classification. Currently, in clinical settings, genetic cascade screening is recommended only when a P/LP variant is found. In addition, a negative test does not imply the exclusion of the genetic cause, as there may be causative genes not yet discovered or the phenotype-genotype relation is still not emphasized. If the genetic test is negative, the first-degree relatives (FDRs) of individuals with the P/LP variant can be reassured and excluded from further clinical cardiac follow-up, resulting in a very efficient management in terms of healthcare-related costs [9,10]; moreover, a negative test also reduces anxiety and uncertainty. If the genetic test is positive, the regular screening with an electrocardiogram (ECG) and echocardiography must be continued to early detect the development of the disease. To make the genetic diagnosis even more difficult, it is important to remember that penetrance and phenotypic expression depends on many factors, such as genetic modifiers, coexistence of multiple causative mutations or environmental contributors. The exact timing for genetic testing in pediatric patients with CMPs is not known in the absence of prospective, large-scale studies due the difficulty of assuring that all ethical principles are applied in this population: autonomy, beneficence, non-maleficence and justice. For a brief insight into the major guideline’s indications referring to the time to start genetic evaluation in CMPs, see Table 1. In the American Heart Failure Society (HFS) [11] there is the recommendation to have a genetic analysis for all affected family members as soon as the diagnosis is made, with the possibility of re-testing, especially in those CMPs, such as DCM, where new rapid panels of genes expand constantly, with the remark that evaluation of infants and children with CMP needs referral to highly specialized centers. An increase as high as 5–10% in sensitivity is considered sufficient to re-test a patient with an inconclusive or negative initial genetic test. Genetic testing is recommended to be started with the subjects most affected as this increases the certitude of the clinical diagnosis and thus of the genetic analysis. In the same guidelines, the mandatory core genes to be included in each CMP group are indicated, and the estimated yield of diagnostic testing is summarized in Table 2. Apart from guidelines, the initiative of the ClinGene group to curate the genes based on pathogenicity evidence should be mentioned, a work still in progress but with excellent clinical utility; according to the group of experts, only genes with definitive or moderate proof of pathogenicity should be used in clinical testing [12]. If genetic testing is inconclusive or not performed in the index and there are no clinical data for CMP, the screening in FDRs is recommended to be done between 10–20 years, every 1–2 years in DCM, HCM and ACM and every 1–3 years in LVNC before 20 years; after 20 years, the European Society of Cardiology (ESC) recommend a screening every 2–5 years for all CMPs [13]; in the American HFS guidelines, the repeated clinical phenotype evaluations should be done every 1–3 years in DCM and ACM and every 2–3 years in HCM and RCM, between 13 to 19 years old [11].

The guidelines, European or American, outline that a genetic diagnosis is merely for a more accurate diagnosis of family members, having less implications for disease management, with some exceptions, e.g., Anderson-Fabry disease for substitution therapy or liver transplantation in transthyretin-related amyloidosis or follow-up for conduction defects and indication for pacemaker in *desmin* (*DES*) and *AMP-activated protein kinase* (*PRKAG2*) mutated subjects.

A statement from the AHA shows that even if almost 40% of children presenting with CMP die or undergo transplantation in the first 2 years, very few of them have a diagnosis, although many of these most probably have a genetic cause [18].

#### 2.1.1. Hypertrophic Cardiomyopathy

HCM is defined in adults as a maximum diastolic wall thickness greater than 15 mm in index cases and 13–14 mm in family members of HCM subjects; in the pediatric population, it is mandatory to report the wall thickness relative to body surface, so the definition is based on wall thickness z-scores greater than two standard deviations from the mean value relative to body surface in an etalon population. It is the second most frequent pediatric CMP, representing 40% of all cases [19].

The majority of causative variants are missense mutations in two genes (*β-myosin heavy chain—MYH7* and *β-myosin binding protein C—MYBPC3*). The ClinGene curation group showed that only 11 genes from 33 HCM genes had moderate or definitive evidence of disease association [20]. The causes of HCM in pediatric subjects are more diverse compared to adults, including mutations in many non-sarcomeric genes triggering storage disorders, neuro-muscular or mitochondrial diseases. Although genetic testing has proved useful for identifying the cause in pediatric CMPs, its use in clinical practice is not a routine, wide-spread tool. The diagnostic yield of genetic testing using a dedicated panel of genes is estimated at 30–60% [21,22,23]. Notwithstanding, genetic testing in HCM can reveal a much more complex genotype with more than one P/LP variant in the same subject, either on both copies of the gene or in different genes. Such genotypes are associated with a younger age of onset (often <10 years), extreme hypertrophy and worse prognosis [24]. Failure to properly identify compound/double heterozygosity negatively affects the management of FDRs, with false reassurance of relatives carrying the unrecognized causative variant. The overall outcome of children with HCM depends on its cause; inborn errors of metabolism have the worst outcome, with roughly 40% survival at 5 years compared with non-infantile idiopathic disease or neuromuscular disorder with a survival >85% at 5 years [25]. From here the idea is given that the identification of the genetic cause gives important information about the prognosis in the short and long term. In phenotype negative individuals, the ESC recommends starting the clinical and/or genetic screening after ten years of age, with earlier screening in cases with malignant/early onset of disease, involvement in competitive sports or presence of symptoms [26]. The ACC/AHA sets the cut-off at an even older age in phenotype negative individuals, between ten and twelve years of age, with the same exceptions referring to competitive sports, family history of SCD or symptomatic subjects, where an earlier genetic test can be undertaken [7]; the genetic testing of any atypical forms of HCM is recommended, as well as for the FDRs of HCM patients. A cohort study showed that as much as a third of children not eligible for early screening by guidelines proved to have HCM [15], suggesting that earlier clinical and genetic evaluations should be made in order to implement lifesaving strategies [27]. Furthermore, another study showed that a significant yield of children diagnosed under 10 years of age received HCM therapies in the form of medications or an internal cardiac defibrillator (ICD), urging a change of paradigm in pediatric genetic testing [28].

Genetic testing to assess the risk of SCD in HCM is seen to be of unknown utility at least in the existing guidelines [7], but more recent, up-to-date studies point out that genetic testing is an excellent tool for prognosis; the presence of sarcomeric mutations increases two-fold the risk for adverse outcomes [29], and furthermore, among sarcomeric variants, carriers of thin filament mutations have a higher risk of developing heart failure [30]. Moreover, troponin mutations are associated with a high risk of SCD despite only mild LVH [31]. Norrish et al. created a risk assessment tool to predict SCD in children with HCM similar to the European version used in adults (HCM Risk-Kids) [32]. However, like many risk calculators, this one needs refinement with genetic data and magnetic resonance markers [33].

The importance of identifying the genetic cause of a pediatric HCM derives also from the existing disease-specific therapies, e.g., enzyme replacement in Pompe disease, lysosomal storage or Fabry disease [34]. The earlier the substitution treatment is initiated, the better the prognosis of the subject.

#### 2.1.2. Dilated Cardiomyopathy (DCM)

Dilatated cardiomyopathy is defined as dilatation and systolic dysfunction of the LV or both ventricles in the absence of coronary artery disease or abnormal filling conditions. A position statement by the ESC also proposes the introduction of the notion hypokinetic (defined as LVEF < 45%) non-dilated cardiomyopathy, as dilation can be absent in some cases [35]. There is a plethora of causes, but for this review we will limit it to the genetic DCM. This is the most encountered CMP in children, representing 50–60% of all cases, with an incidence of 0.57/100.000 children [36]. Thus, DCM is the leading cause of heart transplantation among the pediatric population. Until now, more than 70 genes have been associated with DCM, with *titin* (*TTN*) mutations being the most frequently encountered, in almost one fourth of familial DCM and 18% of non-familial cases. ClinGene’s expert group is currently undertaking a curation evaluation for DCM’s genes that will offer a selection of genes with clinical relevance. Most mutations are inherited autosomal dominant (AD), but all other transmission patterns can be found. The diagnostic yield is relatively low compared with HCM, around 30% with some studies showing a sensitivity as high as 46% [37]. Many confounders impede the genetic testing in DCM, such as the genetic etiology of sporadic DCM or the high genetic heterogeneity of DCM. If there was a level of evidence B for DCM genetic testing in 2013, in the most recent guidelines of the American HFS, the level of evidence is A, due to the numerous studies taken in the last years [11]. Although the American guidelines acknowledge the lack of studies regarding the optimal time to perform the genetic testing, it is advisable that it is done at the time of diagnosis, but can be done also later, whenever available. The same guidelines mention that infants with CMP need special evaluations including genetic testing. A significant number of patients have more than one mutation in more than two genes associated with DCM suggesting an oligogenic inheritance model [35,38]. The American guidelines for genetic evaluation recommend, apart from the DCM core genes, the inclusion of HCM and ACM genes as many genes overlap, while the European guidelines recommend strictly investigating the genes involved in DCM, with the extension only if the family is big enough to have a phenotype-genotype segregation [35].

The family history, premature SCD events or the recommendation of particular therapies play an important role in the decision to undertake genetic testing in DCM [13,39], but these recommendations were made a decade ago when the availability and general knowledge were reduced. Nowadays, guidelines recommend testing all patients with DCM even if there is no family history, as genetic testing may be useful in the general management and risk stratification of family members [11]. One concrete example refers to *laminin* (*LMNA*) mutations, where life threatening arrhythmias can appear despite an LVEF > 35%; from these observations derived the guidelines’ recommendation of primary prophylaxis with an ICD when an *LMNA* mutation is detected, even if LVEF > 35% [11], but also other genes such *DES*, *SCN5A* were associated with high arrhythmogenic risk despite conserved LVEF or mild/moderate dysfunction [5]. DCM patients with variants in the *SCN5A* gene exhibit a phenotype associated with significant arrhythmias and poor response to HF therapy, while treatment with sodium channel blocking drugs reduces the amount of ectopy and normalizes LVEF [40].

#### 2.1.3. Restrictive Cardiomyopathy

Among CMPs, RCM has the lowest prevalence, accounting for only 4.5% of all pediatric cases [19]. From the CMP group, RCM has the poorest prognosis in the short and long term. Regarding the etiology, several diseases are associated with RCM such as amyloidosis, connective tissue diseases, sarcoidosis and inborn errors of metabolism [41], but idiopathic RCM remains the most frequently encountered. The genetics underlying this type of CMP remain mainly unknown, although the most prevalent mutations found were in sarcomeric genes associated with HCM [42], including *troponin I*, *β-myosin heavy chain*, *α-cardiac actin*, *titin* and *myosin light chain*. In addition, *DES* mutations associated with myopathies in general were linked with RCM [43]. The major variability of this CMP even within the same family sustains the idea of multiple genetic factors being involved, including genetic modifiers and numerous interconnected pathways. One interesting study showed that different alternative splicing may influence gene expression in RCM [44]. An important number of studies suggest that RCM and HCM are two phenotypic expressions of the same genetic mutation [45]. Unfortunately, there are no precise indications of when to genetically test a child with RCM, but having seen the poor prognosis and early consideration for transplant, the experts’ opinion is to test as soon as the diagnosis is established [11]. The severity of the disease may be in part explained by the fact that these children have severe pulmonary hypertension more frequently than those with HCM or DCM, an aspect leading some experts to believe that maybe this is a stand-alone disease and not a phenotypic expression of other CMPs [46].

#### 2.1.4. Arrhythmogenic Cardiomyopathy

ACM is an inherited cardiomyopathy defined by progressive fibrofatty replacement of the myocardium, predominantly in the right ventricle [47,48]. The condition is extremely rare among children and very challenging to diagnose before 14 years of age [49,50]. The prevalence of the disease is extremely different depending on the cohort and diagnosis criteria, varying from 5% to almost 30% [51,52]. Evidence concerning pediatric-onset ACM showed that, although rare, the disease may show features starting from childhood, with phenotypic expression comprising life-threatening arrhythmia, heart failure and myocardial inflammation [53]. The cardiac anomalies include right, left or biventricular disease variants with a genetic background related to the phenotypic expression, where RV involvement is associated with the *plakophilin-2* (*PKP2*) gene, LV implication with *desmoplakin* (*DSP*) and *LMNA*, while biventricular expression has a more diverse genetic background. The minimum set of genes to be prioritized in ACM has been recently endorsed by the Heart Rhythm Society (HRS) in their Expert Consesus Statement [17], summarized in Table 2. It should be mentioned that using broad gene panels substantially increases the probability of detecting a VUS (from nearly 10% in an ACM panel to 100% in genome sequencing), while the probability of identifying a disease-causing variant increases only by 10% (from 50% to 60%) [54]. Based on data from 60 heart transplant patients, Chen and colleagues proposed a pathological classification based on circumferential fibrofatty distribution, each of the four clusters having a distinct genetic background [55]. Of note, in cluster one (defined as ‘desmosomal cardiomyopathy’) the mean age of onset was significantly lower compared to the other three clusters (24.74 ± 12.61 years vs 35.63 ± 8.89/30.25 ± 11.01/32.17 ± 11.04). Since the diagnosis criteria are based on adult data, it has been postulated that the clinical work-up in the pediatric population should be revised according to normal ECG and imaging reference values for children [48]. One study showed that echocardiography is very unspecific in the pediatric population with the suggestion of replacement with cardiac magnetic resonance [56]. Once diagnosed, pediatric ACM had a similar clinical disease progression, in terms of sustained ventricular tachycardia, cardiac transplantation and death, as in adults [57]. Still, it is worth mentioning that pediatric patients were more likely to experience SCD or resuscitated sudden cardiac arrest [58,59,60].

#### 2.1.5. Left Ventricular Non-Compaction (LVNC)

LVNC is recognized as a distinct form of cardiomyopathy by the AHA, but not by the World Health Organization or ESC [61]. A wide spectrum of questions is still under debate, such as the accurate definition, if this is a distinct CMP or an additional feature of other CMPs, such as Barth syndrome, the genetic contribution to the development of this phenotype if there is one, the coexistence with other CMPs and the remarkable variations in the expression of the disease, from healthy asymptomatic individuals to severe, pediatric cases. Considered a rare CMP, it probably accounts for approximately 9% of all pediatric cases [62]. In certain cohorts of patients with LVNC mutations were frequently found in same genes as in DCM, such as *TTN* and *LMNA*, suggesting similar pathophysiological mechanisms [63]. All these dilemmas result in a lack of specific indications for genetic screening in LVNC. In the HRS and European Heart Rhythm Association (EHRA) guidelines for genetic testing there is a class IIa indication of genetic testing in LVNC [64], but only a few studies have investigated the yield of genetic testing in pediatric LVNC. There is data showing that idiopathic LVNC has no positive genetic testing, whereas when it co-occurred with other CMP, 12% of individuals tested positive [65]; this observation indicates that genetic testing is only effective if LVNC is connected with features of another CMP (HCM, DCM, RCM or ACM). There is some information that there are more genetic cases in the LVNC pediatric population and that LV systolic dysfunction is a risk factor for major adverse cardiovascular events in mutation carriers [66]. Taken together, despite the lack of multicentric, large studies and uniform guidelines, genetic diagnosis should be undertaken when there are additional red flags such as coexisting CMP, family history of CMP, symptoms or systolic dysfunction. LVNC patients requiring heart transplantation are more frequently pediatric but with similar outcomes after transplantation to patients with idiopathic CMP [67]; this suggests that genetic analysis is necessary as soon as LVNC is associated with the aforementioned red flags.

### 2.2. Channelopathies

Genetic testing recommendations for channelopathies were published in 2011 by Ackerman et al. in a consensus document on behalf of the HRS and EHRA and were reaffirmed in 2018 [64]. In these guidelines, it is clearly stated that the age for genetic testing is disease dependent. For diseases such as LQTS and CPVT where preventive measures exist for genotype positive individuals, the test should be done independent of age, in infancy if available. For other conditions, monitoring the onset of symptoms is recommended rather than discovering a genotype positive-phenotype negative for a disease that may never develop or may develop late (for details see Table 3). The exact time for genetic screening must be rigorously discussed with the family. The diagnostic criteria and management recommendations for patients with hereditary arrhythmic syndromes were initially proposed by a consensus of HRS/EHRA/APHRS in 2013 and revised through the ESC guidelines for SCD prevention in 2015. In the AHA/ACC/HRS guidelines, in patients younger than 40 years of age without structural heart disease, but who experienced unexplained cardiac arrest, recurrent syncope during exertion or unexplained near drowning, genetic testing is seen as important as it may detect an arrhythmia syndrome as etiology of the aforementioned events. Of course, there are other indications for genetic testing, especially in newborns, such as fetal arrhythmias, severe bradycardia, hydrops fetalis or effusions, which could highlight sometimes an underlying CNP, e.g., persistent fetal bradycardia is one of the most frequently encountered manifestation of congenital LQTS [68]. The main CNPs associated with SCD are LQTS, Brugada syndrome (BrS), CPVT and short QT syndrome (SQTS).

#### 2.2.1. Long QT Syndrome

LQTS is a genetic heterogeneous arrhythmic disorder consisting of a typical electrical pattern with long QT intervals and T wave abnormalities, typical polymorphic ventricular tachycardia (VT) (torsade de pointes) and susceptibility for syncope, seizures and SCD at a young age with a structurally normal heart [64]. The estimated incidence is roughly 1:2500; yet, this is possibly underestimated due to asymptomatic cases. LQTS is typically inherited in an AD manner, with an exception being LQTS associated with sensorineural deafness (known as Jervell and Lange-Nielsen syndrome) inherited autosomal recessive (AR). Most individuals diagnosed with LQTS have an affected parent, de novo pathogenic variants being rare.

Considering the high rates of mortality, 50% at 10 years for the index symptomatic cases, underlining that up to 70% of cases may lead to death from the first arrhythmic incident [70], genetic testing is a valuable instrument for early diagnosis, treatment personalization and prospective arrhythmic risk stratification (Table 4). The importance of early disease diagnosis and referral for genetic testing can be illustrated by the results of a prospective study showing that 50% of those with sudden infant death syndrome (SIDS) had a prolonged QT interval during the first week of life [75].

Over 1200 mutations of at least 15 LQTS-causative genes have currently been described (Table 5), but in 75% of cases the disease-causing mutation is found within three “traditional” genes (*KCNQ1*, *KCNH2*, *SCN5A*), accounting for up to 90% of positive genotypes. Only these three traditional genes have sufficient definitive evidence for causation of LQTS [85]. The yield of genetic testing in LQTS varies in the range of 50–86%, being higher in cases with marked QT prolongation and history of SCD [72]. However, negative genetic testing cannot unequivocally exclude LQTS diagnosis by itself; it is noteworthy that up to 25% of patients carrying a pathogenic mutation may have a physiological QTc interval [86]. Interestingly, a study conducted by Miyazaki et al. [69] attempted to evaluate the best age for accurately predicting LQTS using an ECG in children with borderline non-diagnostic QTc (480–499 ms and no unexpected syncope or for QTc < 480 ms with or without syncope). In boys, the period immediately before the onset of puberty, at around 10 years, might be the best time to predict LQTS with borderline LQT intervals, and in girls, at the onset of puberty (12–14 years). Another study showed that life threatening cardiac arrhythmias are rare in pediatric LQTS, where lifestyle modifications and correct beta-blocker therapy were implemented [87]. Furthermore, the most recent AHA/ACC/HRS guidelines for management of patients with ventricular arrhythmia and SCD underline the risk of arrhythmias, especially in infants (<1 year of age) and the importance of early genetic diagnosis [72]. On the other hand, Schwartz et al. [4] recommend the initiation of beta-blocker therapy only in children <1 year with QTc > 470 ms and a follow-up to the age of 1 year when the treatment is re-evaluated and genetic testing is considered, as 40–50% of infants will revert to normal QTc after one year old and genetic testing of individuals with QTc > 470 ms will be negative in 90% of cases. There is a high risk for first cardiac events during adolescence, a crossover in risk by sex at approximately 13 years of age and a lower rate of first cardiac events in adulthood [80]. Importantly, apart from early diagnosis, genetic testing may allow for a genotype-tailored treatment decision in LQTS (Table 4). Beta-blockers are recommended by European guidelines in all patients with clinical diagnosis of LQTS (IB) and should be considered in causative LQTS mutations with normal QT interval. Prospective risk stratification in LQTS may also be assessed by patient genotype particularities which influence the clinical phenotype (Table 4). Arrhythmic episodes (which are bradycardia-dependent and appear at rest) in LQTS-3 have higher lethality than in LQTS-1/2 (roughly 20%), even if events are rarer and happen most frequently before 40 years old [82]. However, in genetically tested individuals, the LQTS-3 genotype is the most powerful predictor of fatal or near-fatal cardiac events after 40 years, warranting the importance of long-term follow-up [84].

#### 2.2.2. Catecholaminergic Polymorphic Ventricular Tachycardia

CPVT is a rare genetic disorder clinically manifested through adrenergically-induced ventricular arrhythmias leading to syncope or SCD during effort or stress at a young age (30% of initial arrhythmic episodes before the age of 10 and 60% before the age of 40) with a structurally normal heart and resting ECG. The mean age of onset of symptoms (usually syncope) is between 7 and 12 years of age [95]. Estimated prevalence is 1:10 000 and the rate of mortality varies in the range of 30–50% before the age of 35 [96]. Even if bidirectional VTs or various polymorphic VTs during effort are typical in CPVT, effort-induced PVCs may be the first sign of the disease. The most frequent form of disease (~65% of cases) is AD (CPVT-1) and consists of mutations in the *ryanodine cardiac RyR2 receptor*, and the rarer form targets the cardiac *calsequestrin* (*CASQ2*) and is AR (3–5%). For rarer forms of CPVT, see Table 5 [90]. More recently, a rare variant in the gene encoding the trans-2,3-enoyl-CoA reductase-like protein (TECRL) was associated with clinical features of CPVT and LQTS [91]. Hayashi et al. [97] demonstrated similar rates of cardiac events in the index CPVT cases versus FDRs subgroup and, moreover, the occurrence of cardiac events in 25% of clinically asymptomatic mutation carrier patients (even during prior effort testing), highlighting the importance of genetic testing and family screening. However, American guidelines have commented that up to 15% of CPVT-diagnosed mutations appear not to be pathogenic, which warrants precaution during genetic testing interpretation [72]. Even so, the European consensus mentions the role of beta-blockers in asymptomatic pathogenic mutation carriers [71], even after a negative exercise test [79].

In first- and second-degree relatives screening (even in the absence of a clinical phenotype) clinical, genetic and when possible exercise stress testing is recommended when diagnosing a pathogenic CPVT mutation in the index case [64]. Canadian guidelines recommend sequential genetic testing starting from *RyR2* mutations, with *CASQ2* testing for *RyR2*-negative cases or in patterns of AR inheritance. A specific subgroup of *RyR2*-negative patients with prominent U waves should be screened for *KCNJ2* mutations [98]. The risk of SCD as the first clinical expression of the disease at a young age (even in infancy, associated with SIDS) justifies genetic screening starting from birth in a family with a known CPVT mutation to allow prompt initiation of prophylactic beta-blockers, with the remark that *CASQ2-CPVT* may be more severe and more resistant to beta-blockers. Efforts for genotype-based risk stratification in CPVT have been made, but current evidence suggests similarity between *CASQ2-* and *RYR2*-related CPVT natural history; Priori et al. [95] and Lehnart et al. [99] have compared affected individuals with and without *RYR2* pathogenic variants and demonstrated a similar age of onset and natural history.

#### 2.2.3. Brugada Syndrome

BrS is a genetic disorder at risk for SCD via polymorphic VT and ventricular fibrillation (VF) on a structurally normal heart with a typical electrical pattern consisting of ST “coved” elevation >2 mm in at least one of V1-V3 spontaneously or facilitated by sodium-channel blockers (such as ajmaline or procainamide) [79]. Prevalence varies between 1:1000 and 1:10 000 with higher ranges in South-East Asia. The mean age of potential lethal arrhythmic events is 41 ± 15 years old and are eight-fold more frequent in men [79].

A “loss-of-function” mutation involving the *SCN5A* gene is most frequently diagnosed in BrS patients (>75% of positive genotypes) [64] and is the only gene with sufficient evidence of pathogenicity [100]. For other pathogenic variants associated with BrS, see Table 5. Less than 35% of clinically diagnosed patients have a positive genotype, most patients remaining genetically elusive. Available data regarding pediatric BrS is scarce, as the natural history of the disease involves clinical and electrical expression during the 4th or 5th decade of life, probably due to the influence of male hormones on phenotypic expression [101], thus making the indications for genetic testing in children extremely difficult to establish in the absence of a family member or appropriate relative with identified BrS-causative mutation, where the genetic testing is recommended as soon as possible [64,72]. However, symptomatic BrS cases have been described in two-day-old infants, and BrS has been associated with SIDS [102,103]. The two largest series on BrS in children included only 30–40 patients [101], and consequently, there is no formal consensus for suspected BrS in infancy and childhood. Children diagnosed through a positive ajmaline test seem to be at low risk, whereas VF during an ajmaline test is frequent in children, which mandates the question of the prospective risk relevance of performing ajmaline tests in asymptomatic children [101]. However, arrhythmic risk is present in children with BrS. In a cohort of 35 children and adolescents with BrS followed up for 88 months, 29% had sustained ventricular arrhythmias, 23% were treated by ICD shocks and 9% had electrical storm [104], justifying an ICD implant in high risk children. Moreover, ICD adverse effects in children are equally important. Corcia et al. [104] showed that 20% of patients had inappropriate shocks (albeit an era effect was observed with a tendency of reduction of such events after 2010), but similarly to adults, symptoms appearance predicted appropriate ICD therapy. Fourteen percent of patients throughout seven years of follow-up had ICD-related complications. However, identifying a disease-causing mutation in a clinically uncertain scenario may help confirm the diagnosis [64], mainly in children where the diagnosis of BrS is difficult [105]. Genetic testing is recommended only in BrS type 1 and not indicated in type 2 or type 3 Brugada ECG patterns [64]. A study querying a prospective registry showed that in subjects ≤ 20 years old with spontaneous or drug-induced Brugada type I ECG pattern and ICD, the rate of appropriate therapy was similar to that of adults [104].

The presence of a *SCN5A* mutation was not associated with arrhythmic events in the FINGER BrS registry [106]. Subsequently, Nishii et al. [107] have shown that even if *SCN5A* mutation is not linked with the occurrence of the first VF episode, it is associated with early and frequent VF recurrence in ICD patients. The limited data on pediatric BrS shows that only a small number of cases exhibit SCD before late adolescence and ECG changes are prompted frequently by fever, therefore, there is a need for aggressive treatment of fever in children with suspicion of BrS, as well as cautious engagement in strenuous activity that increases body temperature above 38 °C. Given that there is only a marginal risk for major cardiac events before 12–15 years of age, the decision to defer genetic testing seems to be reasonable.

#### 2.2.4. Short QT Syndrome

SQTS is an inherited AD heterogeneous arrhythmic disorder leading to syncope and SCD at a young age, especially in infants (up to 4% mortality during the first year of life [94]), with a prevalence in the pediatric population of 0.05% [94]. The probability of a first arrhythmic event is > 40% before 40 years of age and the rate of lethality after a first episode may reach one third of cases. European guidelines proposed a diagnostic QTc ≤ 340 ms or a QTc ≤ 360 ms in the presence of the following: a confirmed pathogenic mutation, a family history of SQTS, a family history of SCD < 40 years or survival from a VT/VF episode in the absence of structural heart disease [79]. Nine genes are currently associated with the SQTS genetic substrate and recommended for sequencing (Table 5), *KCNH2* variants accounting for around 15% of cases. Comprehensive genetic analysis leads to identification of a pathogenic variant in roughly 30% of cases. American guidelines [72] recommend genetic testing in patients with SQTS to facilitate screening of FDRs. There are no available genotype-phenotype correlations permitting risk stratification or treatment personalization recommendations in SQTS [72]. However, patients with SQTS-1 with N588K mutations in the *KCNH2* gene have been shown to have QT prolonging response to quinidine, but no response to class III antiarrhythmic drugs. Although there is paucity in studies, it is considered that class III antiarrhythmic drugs are useful in the rest of the SQTS forms [94,108]. Existing guidelines do not clearly state the recommended age for genetic testing, but since SQTS affects mostly young people, being one of the most common causes of SCD in young athletes, it seems reasonable to perform the test as soon as a clinical suspicion is raised or a family member is diagnosed with SQTS and has a positive genotype [74].

## 3. Patients’ Perspective

While genetic testing is increasingly incorporated into the management of subjects with CMPs and CNPs, little is known about its psychological impact, highlighting the importance of adequate genetic counselling. Some studies investigated the impact of undertaking a cardiogenetic test on patients’ quality of life (QoL) and showed that such an investigation did not influence the health-related QoL in the long and short term in both clinically affected and asymptomatic individuals [109]. A small study in children tested for LQTs, HCM and familial hypercholesterolemia reported positive future perspectives and efficient coping strategies [4]. A study investigating the family perspective on predictive genetic testing for children at risk of LQTS, HCM or ACM pointed out that 92% of respondents believed that testing should be done before 5 years of age for children with LQTS, and 77% reported that genetic testing should be offered before 10 years of age for children at risk of HCM and ACM [110]. On the other hand, other studies showed that subjects with positive genetic tests had increased scores on body pain scales [111] and more intrusive thoughts and distress compared with those testing negative [112]. One study found differences in terms of psychological impact between genetic testing in CMPs and breast cancer, namely that the impact was greater in those with positive genetic testing and no breast cancer [113], whereas in cardiogenetic testing there were no differences between those testing positive based on the presence of symptoms [112,114]. ECG together with genetic testing has a profound stressful impact on parents whose minors undergo predictive testing for LQTS [115]. Hendriks et al. [116] showed that testing for LQTS (ECG and genetic screening) causes distress, especially in carriers with an uncertain ECG and their partners at first visit. Predham et al. [117] evaluated the impact of inconclusive genetic testing results (i.e., VUS or negative result) showing that half of participants with a negative genetic result had an inaccurate perception of what the genetic test result meant for their family members, whilst three main reactions were observed after an inconclusive result: disappointment, questioning and relief. There are few studies that have investigated the impact of genetic testing on children, but one of them showed that gene carriers coped efficiently with the condition, reducing social isolation [118]. On the other side, parents agreeing to genetically test their children did not regret the decision [119]. The majority of studies did not show a negative impact of genetic testing at the psychological level after the announcement of the results [120]. Systematic reviews compared the genetic tests in different fields, cardiovascular, neurodegenerative and oncologic, and showed no increase in distress or QoL, with the exception of Huntington disease [121]. Interestingly, the notion of inheritability of the disease made individuals implanted with ICDs for severe CMP more depressed and with lower QoL compared with those that needed an ICD in the settings of ischemic or valvular disease [122].

For all mentioned situations, genetic counselling before testing is of upmost importance, putting the individual in front of different scenarios and helping them to learn how to face the future. The relevance of genetic counselling is noted in all genetic guidelines as class I indication [17,39]. In children, the preventive genetic testing for diseases that may appear late in life or not at all raises a lot of controversies, especially regarding confidentiality and potential psychological harm, the reason why the American Society of Human Genetics published a position paper in which it recommends deferring genetic testing until an age when the child can make their own decision, unless an early intervention exists [123]. Genetic testing in children should involve a “long-term communication plan for all results” and counselling pre- and post-genetic testing [123]. Future studies with larger pediatric cohorts should be conducted to better understand the different experiences in symptomatic or asymptomatic individuals.

## 4. Conclusions

Genetic diagnosis in pediatric CMPs and CNPs remains a field in continuous development now that the costs of genetic tests have drastically reduced over time. The growing evidence shows that genetic testing has an important influence on the diagnosis, prognosis and treatment of certain CMPs and CNPs, which has increased its use by clinicians, but serious attention should be paid to wise usage and correct interpretation of results, so that patients and family members have the best evaluation. Bench-to-bedside transmission of genetic analysis for pediatric CMPs and CNPs is essential but needs further research and regulations to avoid misinterpretations of results and incorrect decisions, especially in the pediatric population where the decision taken involves not only the patient, but also the family.

## Figures and Tables

**Table 1:** Genetic screening of patients with cardiomyopathies.

Type of Cardiomyopathy	Start of Genetic Evaluation		Additional Comments
	European Guidelines	American Guidelines	
HCM	After 10 years (Class IIa, level of evidence C) [13,14]	After 12 years [7]	Earlier screening in families with malignant early onset disease, presence of cardiac symptoms or involvement in physical activity (Class IIb, level of evidence C) [14]. Younger family members should be considered for early clinical and genetic screening [15]. Australian and New Zealand guidelines recommend the age of 10 years to start the screening [16].
DCM	Childhood (except laminopathies: 10–12 years) [13]	At the time of diagnosis [11]	Genetic screening is questionable in sporadic cases [13].
RCM	10–12 years [13]	At the time of diagnosis [11]	Genetic screening is questionable in sporadic cases [13].
LVNC	New born [13]	Not specified; the decision is guided by the existence of another CMP phenotype, family history of CMP or presence of symptoms [11]	2019 HRS consensus on evaluation of ACM recommends genetic testing (class IIa, level of evidence B) in pathologic LVNC for gene-specific cascade family screening. The American HFS does not recommend the genetic testing in asymptomatic LVNC individuals with normal ventricular function and size.
ACM	10–12 years [13]	10–12 years [17]	ACM has an early adult to adult onset and therefore the 2019 HRS consensus on evaluation of ACM encourages the genetic testing after a certain age when the clinical features are more likely. The screening should start in FDRs at 10–12 years of age.

ACM arrhythmogenic cardiomyopathy, CMP cardiomyopathy, DCM dilated cardiomyopathy, FDR first-degree relative, HCM hypertrophic cardiomyopathy, HFS heart failure society, HRS Heart Rhythm Society; LVNC left ventricular non-compaction, RCM restrictive cardiomyopathy.

**Table 2:** Genotype-phenotype correlation in non-syndromic cardiomyopathies.

Genes	Type of Inheritance	Phenotype					Additional Comments
		HCM	DCM	RCM	LVNC	ACM	
**Sarcomere** **Thick filament**							
MYBPC3	AD	✓	✓	✓	✓		
MYH6	AD	✓	✓				
MYH7	AD	✓	✓	✓	✓		
MYL2	AD	✓		✓			
MYL3	AD, AR	✓		✓			
**Thin filament**							
ACTC1	AD	✓	✓	✓	✓		
TNNC1	AD	✓	✓				
TNNI3	AD, AR	✓	✓	✓			
TNNT2	AD	✓	✓	✓	✓		
TPM1	AD	✓	✓	✓	✓		
**Z-disc**							
ACTN2	AD	✓	✓		✓		
ANKRD1	AD	✓	✓				
BAG3	AD		✓				MM
CSRP3	AD	✓	✓				
LBD3	AD	✓	✓				MM
MYOZ2	AD	✓					
TCAP	AD, AR	✓	✓				LGMD
TTN	AD	✓	✓			✓	
**Desmosome**							
DSC2	AD, AR		✓			✓	
DSFG2	AD		✓			✓	
DSP	AD, AR		✓			✓	Carvajal syndrome (AR)
JUP	AD, AR					✓	Naxos syndrome (AR)
PKP2	AD					✓	Palmoplantar keratoderma
**Intermediate filaments**							
DES	AD, AR		✓			✓	MM
**Nuclear membrane**							
EMD	X-linked		✓				EDMD; LGMD; MM
LMNA	AD, AR	✓	✓				Conduction defects; EDMD
SYNE1	AD		✓				EDMD
**Others**							
CAV3	AD, AR	✓					LGMD; LQTS
CRYAB	AD, AR		✓				MM, cataract
RYR2	AD					✓	
VCL	AD	✓	✓				
PLN	AD	✓	✓				

ACM arrhythmogenic cardiomyopathy; AD autosomal dominant; AR autosomal recessive; CMP cardiomyopathy; DCM dilated cardiomyopathy; EDMD Emery-Dreifuss muscular dystrophy; HCM hypertrophic cardiomyopathy; LGMD limb-girdle muscular dystrophy; LVNC left ventricular non-compaction; MM myofibrillar myopathy; LQTS long QT syndrome; RCM restrictive cardiomyopathy.

**Table 3:** Genetic screening of patients with channelopathies.

Type of Channelopathy	Start of Genetic Evaluation		Additional Comments
	European Guidelines	American Guidelines	
**LQTS**	Infancy [64]	Infancy [64]	Miyazaki et al. [69] study showed that in boys, the period immediately before the onset of puberty (around 10 years) might be the best time to predict LQTS with borderline LQT intervals, and in girls, at the onset of puberty (12–14 years). Schwartz [70] recommends testing after one year of age those with QTc < 485 ms and immediately in all infants with QTc > 485 ms.
**CPVT**	At birth [71]	At birth [72]	
**BrS**	Not specified	Not specified	There are no studies looking at the prevalence and outcomes of asymptomatic pediatric BrS, and the utility of screening for BrS in the juvenile population is low, since the majority will develop electrophysiologic changes in late adolescence. Since the disease has a young-to-adult onset, the start of genetic testing could be deferred after 10–12 years old, or when the child can make their own decision [73].
**SQTS**	Not specified	Not specified	Even if not clearly stated, given the high incidence of SCD in young individuals and athletes, experts recommend genetic testing as soon as possible [74].

BrS Brugada syndrome, CPVT catecholaminergic polymorphic ventricular tachycardia, LQTS long QT syndrome, SCD sudden cardiac death, SQTS short QT syndrome.

**Table 4:** Role of genetic testing in LQTS.

Role	Additional Comments
**Early diagnosis**	The risk of arrhythmias is high in the first year of life [72]. The probability of LQTS-related SIDS may reach 15% of cases [75,76].
**Treatment personalization**	Beta-blocker therapy is superior in LQTS-1 than in LQTS-2 and LQTS-3 [72,77]. LQTS3—modest beta-blocker response [78]—, may require sodium channel blockers such as mexiletine or flecainide [79] or novel agents, e.g., eleclazine. Left cardiac sympathetic denervation (in high-risk patients with recurrent adequate ICD shocks despite maximum tolerated beta-blockers) is more effective in LQTS-1 and LQTS-3 [32,72]. Young LQTS-2 females with QTc > 500 ms are at increased risk of post-partum arrhythmias and may be candidates for a primary prophylaxis ICD or wearable cardioverter defibrillator [72].
**Risk stratification**	In LQTS-1 patients, KCNQ1 mutations involving the transmembrane versus C-terminus region double the risk of arrhythmias [80,81]. KCNQ1 mutations with a functional dominant negative effect (i.e., reduction > 50% in IKs potassium channel activity) versus haploinsufficiency effect (<50% activity reduction) double the risk of arrhythmias. In LQTS-2, *KCNH2* missense mutations involving the transmembrane pore region (S5-loop-S6) have the highest risk of arrhythmias [82]. LQT3 genotype has a 5- to 8-fold higher risk for life-threatening events compared to LQT1 and 2 genotypes [83]. LQTS-3 and ΔKPQ (deletion) mutations of *SCN5A* were shown to be more virulent than those with D1790G mutations [84].

ICD implantable cardiac defibrillator; LQTS long QT syndrome; SIDS sudden infant death syndrome.

**Table 5:** Genes associated with cardiac pediatric channelopathies.

Channelopathy	Genes	References
**LQTS**	KCNQ1—autosomal dominant (LQTS1), KCNH2 (LQT2), SCN5A (LQTS3) (75% cases) Rare: ANK2; KCNE1; KCNE2; KCNJ2; CACNA1C; CAV 3; SCN4B; AKAP9; SNTA1; CALM1; CALM2; KCNJ5; KCNQ1— autosomal recessive (JNL1); KCNE1—autosomal recessive (JNL2)	[88,89]
**CPVT**	CALM1, CASQ2 (68–70% cases) Rare: RYH2; TRDN, TECRL	[90,91]
**BrS**	SCN5A (>75% cases) Rare: PKP2; ABCC9; CACNA1C; CACNA2D1; CACNB2; HCN4; KCND2; KCND3; KCNE3; KCNE5; KCNH2; KCNJ8; RANGRF; SCN1B; SCN2B; SCN3B; SCN10A; SEMA3A; TRM4	[64,92,93]
**SQTS**	KCNH2 (15% cases); CACNA1c; CACNB2; KCNJ2; KCNQ1 Rare: SCN5A; SCL4A3; CACNA2D1; SCN10A	[94]

BrS Brugada syndrome; CPVT catecholaminergic polymorphic ventricular tachycardia; JNL1 Jervell and Lange-Nielsen 1; JNL2 Jervell and Lange-Nielsen 2; LQTS long QT syndrome; SQTS short QT syndrome.
